# Efficacy of Yishen Huashi Granules Combined with Linagliptin Tablets on Blood Glucose and Renal Function in Patients with Type 2 Diabetic Nephropathy

**DOI:** 10.1155/2022/4272520

**Published:** 2022-09-20

**Authors:** Panke Zhang, Jingxi Meng, Mingliang Duan, Dan Li, Ruixin Wang

**Affiliations:** ^1^Department of Nephrology Rheumatology, Zhengzhou Hospital of Traditional Chinese Medicine, Zhengzhou 450000, Henan, China; ^2^Department of Diabetes, Zhengzhou Hospital of Traditional Chinese Medicine, Zhengzhou 450000, Henan, China

## Abstract

**Objective:**

To probe into the efficacy of Yishen Huashi granules combined with linagliptin tablets in the treatment of type 2 diabetic nephropathy (DN) and its effect on blood glucose and renal function in patients.

**Methods:**

70 patients with type 2 DN at our hospital between May 2020 and May 2022 were chosen as the research objects and separated into the control group and the research group based on their treatments. With 35 cases in each group, the patients treated with initial therapy and linagliptin tablets were enrolled in the control group, and those who received the above treatments and also Yishen Huashi granules were included in the research group. Their clinical indexes such as blood glucose and renal function were compared with both groups after treatment.

**Results:**

After treatment, the research group had remarkably lower fasting blood glucose (FPG), 2 h-postprandial blood glucose (2 h-PBG), and glycosylated hemoglobin A1c (HbA1c) levels than those in the control group (*P* < 0.05). After treatment, the research group had remarkably lower levels of total cholesterol (TC), triglyceride (TG), and low-density lipoprotein (LDL) (*P* < 0.05) and higher high-density lipoprotein (HDL) levels (*P* < 0.05) than those in the control group. After treatment, the urinary microalbumin (u-mALB) level was remarkably lower in both groups (*P* < 0.05) and was distinctly lower in the research group than in the control group (*P* < 0.05). After treatment, the research group had remarkably lower renal function indexes such as serum creatinine (SCr), blood urea nitrogen (BUN), urinary protein (UPro), and urinary albumin excretion rate (UAER) (*P* < 0.05) and a higher estimated glomerular filtration rate (eGFR) level (*P* < 0.05) than those in the control group. The efficacy was evaluated by the traditional Chinese medicine (TCM) syndrome score after treatment. There were no patients in complete remission between both the groups, where slight differences were found in the proportion of significant remission (*P* > 0.05), with the total effective rate of the research group remarkably higher than that of the control group (*P* < 0.05).

**Conclusion:**

The combination of Yishen Huashi granules and linagliptin tablets can reduce the blood glucose and blood lipid levels in patients with type 2 DN and lower UPro and protect renal function at the same time, which provides a new idea and a method for clinical treatment of type 2 DN with integrated traditional Chinese and Western medicine.

## 1. Introduction

Diabetic nephropathy (DN), characterized by persistent proteinuria, decreased glomerular filtration rate (GFR), and elevated blood pressure, is the major primary disease of end-stage nephropathy and one of the lethal microvascular complications of diabetes, with an incidence rate reaching up to 30%–50% [1–3]. DN has an insidious onset, which progresses rapidly by the proteinuria stage in the clinic, with symptoms such as edema, dyslipidemia, and continuous decline in renal function. However, conventional hypoglycemic agents are limited in DN treatment, and most oral hypoglycemic agents cannot be used in stages 3–4 of chronic kidney disease (CKD) [4–6]. In contrast, as a new type of a dipeptidyl peptidase 4 (DPP-4) inhibitor, linagliptin tablets can be excreted through the intestine and can be used even in patients with renal insufficiency or on dialysis, with a good hypoglycemic effect. In addition, traditional Chinese medicine (TCM) treatment has been applied to DN and CKD for more than 2,000 years, whose herbal medicine is still widely adopted in treating DN conditions. From the perspective of TCM, DN is a syndrome of qi and yin deficiency with blood stasis, whose treatment should be based on nourishing qi and yin, activating blood circulation, and removing blood stasis. Yishen Huashi granules, which are composed of ginseng, astragalus, large head atractylodes rhizome, Poria cocos, Rhizoma Alismatis, ternate pinellia rhizome, notopterygium root, radix angelicae tuhuo, divaricate saposhnikovia root, radix bupleuri, etc., have the effect of invigorating yang and spleen, tonifying kidneys, dissipating hygrosis, and inducing diuresis and detumescence. Pharmacological studies have also confirmed that the granules can reduce UPro, regulate immune function, inhibit oxidation, reduce glycemic indexes, and repair damaged glomerular basement membranes [7–9]. So far, there has been no research combining these two in the treatment of DN. In our hospital, we have been using Yishen Huashi granules in combination with linagliptin tablets to treat type 2 DN for many years with good efficacy, which is reported as follows.

## 2. Materials and Methods

### 2.1. Inclusion Criteria

① The patients' disease met the clinical diagnostic criteria of type 2 DN [10] and was stable; ② the patients were aged 18 and older; ③ the patients' gender was not limited; ④ the patients had good compliance with treatment and follow-ups; ⑤ the patients did not take any vitamins, statins, or angiotensin-converting enzyme inhibitors within the last 30 days; ⑥ the patients' disease met the indications for Yishen Huashi granules and linagliptin tablets;⑦ the patients and their family members knew the purpose and procedure of this study and signed the informed consent.

### 2.2. Exclusion Criteria

① Patients with severe liver and kidney dysfunction, malignant tumor, or coagulation disorders; ② patients receiving maintenance dialysis or renal transplantation; ③ patients with other primary or secondary nephropathy; ④ patients with diabetic ketoacidosis; ⑤ patients with hyperthyroidism or severe infection;⑥ patients with renovascular hypertension.

### 2.3. Selection and Grouping of Patients

70 patients with type 2 DN at our hospital between May 2020 and May 2022 were chosen as research objects to conduct a retrospective analysis. They were separated into a control group and a research group based on the treatment methods, with 35 cases in each group. This study conformed to ethical standards and was authorized by the ethics committee of our hospital.

### 2.4. Treatment Methods

Patients were all given conventional treatment of DN, including high-quality-low-protein, low-phosphorus diabetic diet, control of blood glucose and blood pressure, correction of anemia, correction of acidosis, correction of disorders of calcium and phosphorus metabolism, regulation of blood lipids, maintenance of electrolyte balance, and prevention and control of infection [11–13]. Based on this, the control group took linagliptin tablets (specification: 5 mg; manufacturer: Boehringer Ingelheim Pharmaceuticals Inc.; NMPA Approval No. J20171087) orally, 5 mg per day, which could be taken at any time of the day, with or without meals. With the above treatments, the research group took Yishen Huashi granules (specification: 10 g/bag; manufacturer: Guangzhou Consun Pharmaceutical Co., Ltd.; NMPA Approval No. Z20090250), 1 bag each time, 3 times a day. The observation treatment period of both groups was 3 months.

### 2.5. Observation Criteria

1. General data include age, gender, duration of diabetes, concomitant risk factors, body mass index (BMI), fasting blood glucose (FPG), 2 h-postprandial blood glucose (2 h-PBG), glycosylated hemoglobin A1c (HbA1c), total cholesterol (TC), triglyceride (TG), serum creatinine (SCr), blood urea nitrogen (BUN), tumor necrosis factor-*α* (TNF-*α*), interleukin-6 (IL-6), high-sensitivity C-reactive protein (hs-CRP). SCr is generally considered to be endogenous SCr, while endogenous creatinine is a product of human muscle metabolism. BUN is a nitrogenous compound in plasma other than protein, which is excreted from the body by glomerular filtration. BUN will increase when renal insufficiency is decompensated. The presence of protein in the urine is called proteinuria or UPro. Normal urine contains a small amount of small molecule protein, and proteinuria is a common manifestation of kidney disease and can also occur in systemic diseases.
2. Glycemic indexes. The glycemic indexes of patients in both groups were measured after treatment, including FPG, 2h-PBG, and HbA1c indexes.
3. Blood lipid levels. The blood lipid levels in the two groups were tested after treatment, including TC, TG, high-density lipoprotein (HDL), and low-density lipoprotein (LDL) indexes.
4. Urinary microalbumin (u-mALB). The u-mALB levels were tested before and after treatment in patients.
5. Renal function includes SCr, BUN, estimated GFR (eGFR), urinary protein (UPro), and urinary albumin excretion rate (UAER) indexes.
6. Efficacy in treating DN assessed by the TCM syndrome score. Patients who had negative results of proteinuria, UPro quantification of <0.5 g/24h, normal or near normal serum albumin levels (>35 g/L), normal or near normal urinary erythrocyte levels, normal renal function, and complete elimination of clinical nephropathic symptoms by multiple detections were complete remission. Patients who had UPro quantification of <1 g/24h, remarkably improved serum albumin and urinary erythrocyte levels, and normal or near normal renal function by multiple detections were significant remission. Multiple detection results of decreased UPro levels, UPro quantification reduced by half compared with that before treatment, improved serum albumin and urinary erythrocyte levels, and better renal function were partial remission. UPro, serum albumin, and urinary erythrocyte levels had no remarkable changes compared with those before treatment, with obvious clinical nephropathic symptoms and unchanged renal function as invalid treatment.

#### 2.5.1. Laboratory Testing

Venous blood samples were taken from patients before and after treatment for laboratory testing, with the indexes of FPG, HbA1c, TC, TG, SCr, BUN, HDL, LDL, TNF-*α*, IL-6, and hs-CRP. 24-h urine samples were collected before and after treatment, 50 ml of which were taken for testing, with the indexes of u-mALB, eGFR, UPro, and UAER.

### 2.6. Statistical Disposal

The study adopted SPSS22.0 for data processing, which mainly calculated the differences between the groups, and graph production was carried out using GraphPad Prism 7 (GraphPad Software, San Diego, USA). The research data contained two types of data, count data and measurement data, in which the former was represented as [*n* (%)] and verified by *X*^2^ and the latter was represented as ($\bar{x}\pm s$) and verified by *t* tests, conformed to normal distribution. The statistical result of *P* < 0.05 indicated a statistical difference between the groups.

## 3. Results

### 3.1. General Data

No statistical differences were found in the age, genders, duration of diabetes, concomitant risk factors, BMI, FPG, 2h-PBG, HbA1c, TC, TG, SCr, BUN, TNF-*α*, IL-6, and hs-CRP between both the groups (*P* > 0.05). The general data in both groups were balanced and comparable, see Table 1.

### 3.2. Glycemic Indexes

The glycemic indexes of all the patients showed reductions after treatment compared to those before treatment, with the FPG, 2 h-PBG, and HbA1c levels remarkably lower in the research group than those in the control group (*P* < 0.05), see Table 2.

### 3.3. Blood Lipid Levels

After treatment, the research group had remarkably lower TC, TG, and LDL levels (*P* < 0.05) and higher HDL levels (*P* < 0.05) than those in the control group. The blood lipid levels in the research group after treatment were eminently better than those in the control group, see Table 3.

### 3.4. U-mALB Levels

**Figure 1:**
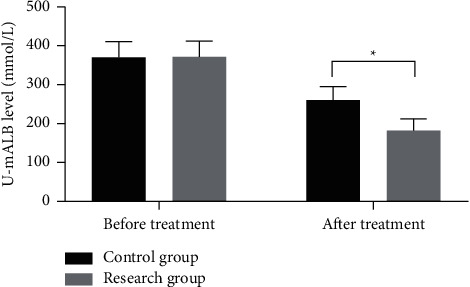
Comparison of u-mALB levels. Notes: the transverse axis was before and after treatment, with the longitudinal axis as the u-mALB level (mmol/L). The u-mALB levels in the control group before and after treatment were (370.37 ± 40.36) and (260.78 ± 34.24), respectively, while in the research group were (371.45 ± 40.58) and (182.13 ± 30.56). ^*∗*^ indicated a remarkable difference in the u-mALB levels between both the groups after treatment (*t* = 10.138, *P* < 0.001).

After treatment, the u-mALB level was remarkably lower in both groups (*P* < 0.05) and was distinctly lower in the research group than in the control group (*P* < 0.05), see Figure 1.

### 3.5. Renal Function

After treatment, the research group had remarkably lower renal function indexes such as SCr, BUN, UPro, and UAER (*P* < 0.05) and a higher eGFR index (*P* < 0.05) than those in the control group, see Table 4.

### 3.6. Efficacy  of TCM Syndrome

**Figure 2:**
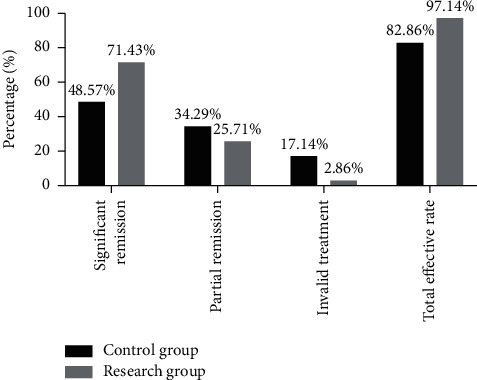
Comparison of efficacy of TCM syndrome after treatment (*n* = 35). Notes: the transverse axis represented evaluation dimensions, with the longitudinal axis as the percentage (%). After treatment, there were 17 cases of significant remission, 12 cases of partial remission, 6 cases of invalid treatment, and a total of 29 cases of effective treatment in the control group; there were 25 significant remissions, 9 partial remissions, 1 invalid treatment, and a total of 34 effective treatment in the research group. ^*∗*^ suggested a remarkable difference in the total effective rate of treatment between both the groups (*X*^2^ = 3.968, *P*=0.046).

The efficacy was evaluated by the TCM syndrome score after treatment. There were no patients in complete remission between both the groups, where slight differences were found in the proportion of significant remission (*P* > 0.05), with the total effective rate of the research group being remarkably higher than that of the control group (*P* < 0.05), see Figure 2.

## 4. Discussion

Type 2 DN is a microvascular complication often taking place in diabetic patients, with insidious onset, less obvious initial symptoms, and malignant changes in renal function, when the disease develops to the late stage. Diabetes is the major factor of end-stage renal disease, and a clinical study has confirmed [14] that DN occurs in most patients 15–20 years after diabetes, which is one of the lethal hazards to diabetic patients. However, in recent years, TCM treatment has been widely carried out in clinics, bringing more opportunities and possibilities for treating type 2 DN. TCM believes that DN belongs to the categories of “edema” and “guange” (frequent vomiting and dysuria), which is developed on the basis of the deficiency of qi and yin in diabetes and obstruction of collaterals by blood stasis and toxin. The pathogenesis is mainly the deficiency of the spleen and the kidney, incompetence of qi transformation, and internal resistance of blood stasis [15, 16]. Due to long-term diabetes and weak qi of the spleen, the source of qi and blood is blocked, resulting in the kidney's failure of storing energy and dysfunction of water metabolism. Ginseng, large head atractylodes rhizome, and Poria cocos in Yishen Huashi granules have the effect of invigorating yang and qi and strengthening the spleen to eliminate dampness, which can remove the influence of damp in middle jiao on the kidney. *Pinellia ternata* rhizome, rhizoma alismatis, and divaricate saposhnikovia root enable the recovery of renal function by eliminating dampness and swelling and tonifying qi and the kidney. Rhizoma coptidis, the root of herbaceous peony, and dried old orange peel can remove dampness. For DN, there are limitations in the treatment of Western medicine or TCM alone. Most conventional oral hypoglycemic agents in the clinic need to be excreted by the kidneys, and thus, most patients need to receive insulin therapy. But as renal clearance of insulin decreases and the duration of insulin action prolongs, the risk of hypoglycemia gradually increases, and even cardiovascular and cerebrovascular events can be induced [17, 18]. Linagliptin is a xanthine derivative which has the advantages of strong activity, high DPP-4 receptor selectivity, long half-life, and a high protein binding rate. Most of the drug is excreted via the intestinal tract as a prototype, with only 5% by the kidney. It is the only DPP-4 inhibitor that does not require dose adjustment in the treatment of DN, which has good hypoglycemic effects even in CKD stage 5 and can continue to be used in the presence of hepatic insufficiency [19, 20]. The combined application of Linagliptin and Yishen Huashi granules may create synergy, which is beneficial to improve the clinical efficacy of DN patients.

### 4.1. Effect on Glycemic Indexes and Blood Lipid Levels

The results revealed that all the patients' glycemic indexes showed different degrees of reduction after treatment compared to those before treatment, with the FPG, 2hPG, and HbA1C levels in the research group remarkably lower than those in the control group (*P* < 0.05). After treatment, the research group had remarkably lower TC, TG, and LDL levels (*P* < 0.05) and higher HDL levels (*P* < 0.05) than those in the control group, suggesting eminently better glycemic indexes and blood lipid levels in the research group after treatment than those in the control group. Previous studies have shown [21–23] that Yishen Huashi granules can control blood glucose and regulate blood lipids. From the standpoint of TCM, hyperlipidemia belongs to the category of “stagnation of damp turbidity and static blood,” which is mainly treated by invigorating yang and discharging turbidity, activating blood circulation, and removing blood stasis. Yishen Huashi granules have the functions of invigorating yang and the spleen, eliminating dampness and turbidity, and inducing diuresis and detumescence. According to modern pharmacology, astragalus can improve blood microcirculation, and rhizoma alismatis can inhibit the occurrence of hyperglycemia and hypersensitivity, thus stabilizing blood glucose and blood lipids and reducing the occurrence of glomerular basement membrane lesions. Therefore, Yishen Huashi granules can effectively alleviate the symptoms and signs of edema, fatigue, inappetence, chillness, and cold limbs caused by DN.

### 4.2. Effect on Renal Function

According to the analysis on the renal function-related indexes of the two groups, the u-mALB level was remarkably lower in both groups after treatment (*P* < 0.05) and was distinctly lower in the research group than in the control group (*P* < 0.05). After treatment, the research group had remarkably lower renal function indexes such as SCr, BUN, UPro, and UAER (*P* < 0.05) and a higher eGFR index (*P* < 0.05) than those in the control group, suggesting that the combination of Yishen Huashi granules and linagliptin had a better effect on improving renal function and reducing renal injury. Modern pharmacology has shown that ginsenosides in Yisheng Huashi granules can reduce proteinuria, diminish inflammation and water retention, and protect renal function; polysaccharides in Poria cocos activate the cellular immune response and improve patients' low immunity, thereby alleviating the inflammatory response and promoting the repair of renal tubular damage [24, 25]. With the addition of linagliptin tablets, this combination can exert renal protective effects while lowering blood glucose. Moreover, the efficacy evaluated by the TCM syndrome score after treatment showed that there were no patients in complete remission between both the groups, where slight differences were found in the proportion of significant remission (*P* > 0.05), with the total effective rate of the research group remarkably higher than that of the control group (*P* < 0.05). It has further confirmed that Yishen Huashi granules combined with linagliptin tablets have a good effect of glucose control on patients with type 2 DN and contributes to the reduction of renal injury, with a definite curative effect and further promoting the rehabilitation of patients.

## 5. Summary

The combination of Yishen Huashi granules and linagliptin tablets can effectively reduce blood lipids and control blood glucose, with the effect of lowering UPro and protecting renal function, which provides a new idea and a method for clinical treatment of type 2 DN with integrated traditional Chinese and Western medicine. It is speculated in the study that lipid lowering may have an obvious effect on controlling UPro, improving hypercoagulability, and delaying the disease progression of DN patients. But it is worth noting that the sample size selected for this study was limited and that the findings may be influenced by factors such as geographical factors, so the correlation can be confirmed by the forward large-sample studies.

## Figures and Tables

**Table 1:** Comparison of general data (*n* = 35).

Observation indexes	Control group	Research group	*X * ^2^/*t*	*P*
Age (years)	58.51 ± 8.30	58.34 ± 8.40	0.085	0.932
Genders			0.238	0.626
Male	20 (57.14)	22 (62.86)		
Female	15 (42.86)	13 (37.14)		

Duration of diabetes (years)	5.89 ± 1.12	6.00 ± 1.15	0.405	0.687
FPG (mmol/L)	10.25 ± 0.67	10.34 ± 0.71	0.545	0.587
2h-PBG (mmol/L)	12.56 ± 1.97	12.50 ± 1.88	0.130	0.897
HbA1c (%)	8.80 ± 1.47	8.78 ± 1.50	0.056	0.955
TC (mmol/L)	5.47 ± 0.65	5.48 ± 0.53	0.071	0.944
TG (mmol/L)	2.08 ± 1.40	2.13 ± 0.38	0.204	0.839
SCr (umol/L)	73.05 ± 16.85	72.96 ± 17.10	0.22	0.982
BUN (mmol/L)	8.53 ± 5.59	8.47 ± 5.41	0.046	0.964

Concomitant risk factors				
Hypertension	29 (82.86)	31 (88.57)	0.467	0.495
Hyperlipidemia	20 (57.14)	19 (54.29)	0.058	0.810
BMI (kg/m^2^)	20.86 ± 2.01	21.05 ± 2.10	0.387	0.700
TNF-*α* (ng/L)	141.84 ± 24.10	142.48 ± 23.71	0.112	0.911
IL-6 (ng/L)	60.07 ± 8.37	60.20 ± 8.85	0.063	0.950
Hs-CRP (mg/L)	7.55 ± 1.85	7.48 ± 1.83	0.159	0.874

**Table 2:** Comparison of glycemic indexes after treatment.

Groups	Number of cases	FPG (mmol/L)	2 h-PBG (mmol/L)	HbA1C (%)
Control group	35	8.70 ± 0.90	11.07 ± 1.97	8.55 ± 0.70
Research group	35	7.33 ± 0.80	10.10 ± 1.65	7.46 ± 0.68
*t*		6.731	2.233	6.608
*P*		<0.001	0.029	<0.001

**Table 3:** Comparison of blood lipid levels after treatment (*n* = 35).

Groups	TC (mmol/L)	TG (mmol/L)	HDL (mmol/L)	LDL (mmol/L)
Control group	4.70 ± 0.27	2.02 ± 0.26	1.03 ± 0.08	3.18 ± 0.29
Research group	4.23 ± 0.24	1.85 ± 0.22	1.16 ± 0.13	2.51 ± 0.16
*t*	6.697	2.953	5.038	11.968
*P*	<0.001	0.004	<0.001	<0.001

**Table 4:** Comparison of renal function indexes after treatment (*n* = 35).

Groups	SCr (umol/L)	eGFR (ml/min·1.73 m^2^)	BUN (mmol/L)	UPro (g/24h)	UAER (ug/min)
Control group	67.33 ± 8.14	80.64 ± 6.46	7.21 ± 2.15	1.15 ± 0.22	76.15 ± 10.72
Research group	62.12 ± 6.93	86.12 ± 10.96	6.23 ± 1.55	1.00 ± 0.18	70.83 ± 8.34
*t*	2.883	2.548	2.187	3.122	2.317
*P*	0.005	0.013	0.032	0.03	0.024

## Data Availability

Data used to support the findings of this study are available on reasonable request from the corresponding author.
